# Effects of high-fiber food on gut microbiology and energy metabolism in *Eothenomys miletus* at different altitudes

**DOI:** 10.3389/fmicb.2023.1264109

**Published:** 2023-08-31

**Authors:** Wei Zhang, Ting Jia, Hao Zhang, Wanlong Zhu

**Affiliations:** ^1^Key Laboratory of Ecological Adaptive Evolution and Conservation on Animals-Plants in Southwest Mountain Ecosystem of Yunnan Province Higher Institutes College, School of Life Sciences, Yunnan Normal University, Kunming, China; ^2^Engineering Research Center of Sustainable Development and Utilization of Biomass Energy Ministry of Education, Kunming, China; ^3^Key Laboratory of Yunnan Province for Biomass Energy and Environment Biotechnology, Kunming, China

**Keywords:** *Eothenomys miletus*, intestinal microbiota, high-fiber foods, body mass regulation, adaptation

## Abstract

Intestinal microorganisms assist the host in digesting complex and difficultly decomposed foods; expand the host’s dietary ecological niche. In order to investigate the effect of high-fiber food on intestinal microorganisms of *Eothenomys miletus* at different altitudes, exploring the regional differences of intestinal microorganisms and their roles in body mass regulation, we collected *E. miletus* from Dali (DL) and Xianggelila (XGLL), which were divided into control group, high-fiber group fed with high-fiber diet for 7 days, and refeeding group fed with standard diet for 14 days after high-fiber diet. Using 16S rRNA gene sequencing technology combined with physiological methods, we analyzed the gut microbial diversity, abundance, community structure and related physiological indicators of each group, and explored the effects of high-fiber foods and regions on the diversity, structure of gut microorganisms and physiological indicators. The results showed that high-fiber food affected the food intake and metabolic rate of *E. miletus*, which also showed regional differences. The intestinal microorganisms of *E. miletus* obtained energy through the enrichment of fiber degrading bacteria under the condition of high-fiber food, while producing short-chain fatty acids, which participated in processes such as energy metabolism or immune regulation. Moreover, it also affected the colonization of intestinal microorganisms. High-fiber food promoted the enrichment of probiotics in the intestinal microbiota of *E. miletus*, but pathogenic bacteria also appeared. Therefore, the changes in the composition and diversity of gut microbiota in *E. miletus* provided important guarantees for their adaptation to high fiber food environments in winter.

## 1. Introduction

Gut microorganisms, which are numerous and diverse, are the main symbiotic taxa of mammals, and they not only obtain nutrients from their hosts, but also have a profound impact on individual’s development, nutrient acquisition, physiological functions, immune regulation and other important activities (Dillon et al., 2005; Ezenwa et al., 2012; Sender et al., 2016). During the long-term evolutionary process, stable mutual adaptation and collaboration relationships have been formed between animals and their gut microbiota, and co-evolution has been achieved (Zhang et al., 2016). The animal gut microbiota possesses millions of evolutionarily distinct gene families, some of which can assist the host in obtaining indigestible food and expanding the hosts’ metabolic capacity (Xiao et al., 2015; Moeller and Sanders, 2020). In herbivores, the complex polysaccharides contained in plants could not be digested by animal enzymes alone, but microorganisms in the gut can ferment these compounds, *Lactobacillus*, for example, *Ruminococcus*, *Eubacterium*, and *Fusobacterium*, amongst others, can ferment different carbohydrates to produce short-chain fatty acids (SCFAs) and other metabolites that were readily available to the host (Salyers et al., 1977). SCFAs (such as acetate and butyrate) provide much of the energy required to maintain high turnover rates of colonocytes and intestinal epithelial cells in the intestine, with oxidation of butyrate alone able to provide 70% of the energy required by colonocytes in rat (Roediger, 1982; Moeller and Sanders, 2020). Moreover, gut microbiota also enhanced the digestion of plant secondary metabolites by herbivorous animals. For example, *Neotoma lepida* feed on juniper and leguminous shrubs rich in toxic creosote, and experiments have demonstrated that gut microorganisms such as *Enterococcus*, *Clostridium*, and *Lactobacillus* increased the tolerance and digestibility of creosote secondary metabolites in forest rats (Kohl and Dearing, 2016). It was found that when *Neotoma lepida* was fed a diet containing creosote, microorganisms with genes associated with aromatic compound metabolism were selected for in the gut microbial community, and there was an increase in the abundance of enzymes associated with the metabolism of aromatic compounds, for instance, aryl-alcohol dehydrogenase, which helps the host to degrade toxic components of plant secondary metabolites like aromatic compounds (Kohl et al., 2014). Intestinal microorganisms help the host digest complex and difficultly decomposed food, expand the host’s dietary ecological niche, affected its ability to compete for scarce resources, and enhance its adaptation to the environment (Gilbert et al., 2015; Alberdi et al., 2016).

Acquisition of energy from food and its energy distribution is an important factor in the survival, abundance or distribution in animals (Veloso and Bozinovic, 1993). However, wild small mammals were likely to experience a decline in food quality during their life cycle, such as immature seeds, winter or early spring when vegetations were not abundant, forcing them to feed on high-fiber foods, but the efficiency of obtaining energy from high-fiber foods was relatively low (Bozinovic, 1995). The host’s gut microbiota increased the proportion and diversity of cellulose degrading microorganisms by altering their diversity and composition, promoting the host’s utilization of high-fiber foods, and thus maintaining the host’s energy balance (Varel and Dehority, 1989; Ni and Tokuda, 2013; Lindsay et al., 2020). For example, pigs on a high-fiber diet for a short period of time had an altered intestinal microbiota, in particular an increasing in the abundance of dominant fiber-degrading bacteria, which digested more fiber to provide energy, and an increasing in the proportion of fiber in the diet lead to the fecal higher abundance of cellulose- and starch-fermenting bacteria in *Rhinopithecus roxellanae* (Liu et al., 2013; Pu et al., 2020). Meanwhile, the increasing of fiber content in food contributes to the intestinal health of animals, such as the production of volatile fatty acids and other metabolites, promoting the production of probiotics such as Bacteroides, and maintaining the micro balance of gut microbiota (Russell and Duthie, 2011; Geirnaert et al., 2012; Scott et al., 2013). Researches results also indicating that excessive dietary fiber lead to an increasing in the abundance of pathogenic bacteria (Pu et al., 2023). At present, there were various studies on the impact of high-fiber foods on gut microbiota, and there was no consensus on the changes in host physiology and gut microbiota under high-fiber foods.

Hengduan Mountains are located in southwestern China, in the southeastern part of the Qinghai-Tibetan Plateau, which is characterized by decreasing temperatures and increasing precipitation with increasing altitude, with distinct dry and wet seasons, small annual temperature differences and large daily temperature differences (Gong et al., 2001; Wang et al., 2017). *Eothenomys miletus* is a native species of Hengduan Mountains (Lou et al., 2000). *E. miletus* mainly feed on plants rich in high-fiber, such as Poaceae, and the food diversity of *E. miletus* at higher altitude was higher (Yan and Zhu, 2023). Previous studies have shown that temperature, photoperiod and food were important environmental factors affecting the energy metabolism in *E. miletus*, and there were significant differences in their body mass when facing different food treatments (Zhu et al., 2010, 2011; Mu et al., 2015). *E. miletus* in different regions adapts to different environments by regulating body mass, liver, digestive tract, other body composition and related hormone expressions (Han et al., 2021). Moreover, our studies have confirmed that there were differences in the structure and diversity of the intestinal bacterial community of *E. miletus* in different regions, which was a positive response to changes in food and environment (Yan et al., 2022). The present study used 16S rRNA gene sequencing technology, combined with relevant physiological indicators, to investigate the differences in the effects of high-fiber food on gut microorganisms and body mass regulation of *E. miletus* at different altitudes (Dali, DL and Xianggelila, XGLL) in winter, and ultimately to elucidate the relationship between gut microorganisms and its body mass regulation. We hypothesized that high fiber foods will affect the diversity and composition of gut microbiota, with regional differences of regulation for gut microbiota in *E. miletus*.

## 2. Materials and methods

### 2.1. Collection of experimental animals

Samples were collected in DL and XGLL in winter of 2022. *E. miletus* were all healthy adult individuals with non-reproductive periods. The geographical location, climate characteristics, and number of the sampling points were detailed in Table 1.

**TABLE 1:** Geographical locations and main conditions for two regions of *Eothenomys miletus.*

Regions	Sample number	Body mass (mean ± SE)	Longitude and latitude	Altitude/m	Mean winter temperature/°C	Precipitation/ mm	Vegetation type
XGLL	20 (10♂/10♀)	36.45 ± 2.32	99°83′16″ E, 27°90′73″ N	3321	4.5	984.2	Subalpine meadows
DL	21 (11♂/10♀)	32.70 ± 1.65	100°42′49″ E, 24°90′30″ N	2217	17.5	597.0	Savannah shrubs

XGLL, Xianggelila; DL, Dali.

### 2.2. Treatment of experimental animals

After disinfection and flea killing, the captured *E. miletus* from the two regions were taken back to the animal breeding room of Yunnan Normal University, and were housed singly in a mouse box (260 mm × 160 mm × 150 mm). After 4 days of laboratory adaptation, a two-factor (region × high-fiber food) experimental design was used, and *E. miletus* were divided into 0-day control group, 7-day high-fiber group, and 14-day refeeding group after the high-fiber diet followed by refeeding with standard diet. That is, Dali control group (DLC, *n* = 7), Dali high-fiber group (DLHF, *n* = 7), Dali refeeding group (DLRe, *n* = 7), Xianggelila control group (XGC, *n* = 7), Xianggelila high-fiber group (XGHF, *n* = 7), Xianggelila refeeding group (XGRe, *n* = 6). Room temperature was controlled at 25 ± 1°C and the photoperiod was 12L:12D (Light: Dark). Animals were fed standard rat chow and high-fiber chow (produced by Kunming Medical University), and the food composition is shown in Table 2. Food and water were provided *ad libitum*. The experiment lasted for 21 days, with body mass, food intake, and resting metabolic rate (RMR) measured on day 0, 7, and 21. Body mass was measured on an LT502 electronic balance (accurate to 0.01 g), food intake was measured by the food balance method and RMR was measured by a portable respirometer, as described by Zhu et al. (2014) and Gong et al. (2022). After the determination of relevant indicators, pentobarbital sodium (50 mg/kg) was used for anesthesia and execution, and serum and rectal feces were taken.

**TABLE 2:** Different food components.

Contents	Standard diet	High-fiber diet
Crude fat (%)	6.2	3.9
Crude protein (%)	20.8	19.4
Neutral detergent fiber (%)	21.5	35.5
Acid detergent fiber (%)	12.5	21.4
Ash (%)	10.0	10.5
Caloric value (kJ/g)	17.5	17.3

### 2.3. Determination of physiological indicators

Blood was taken at the end of each group’s experiment by execution, and the blood was rested in a refrigerator at 4°C for 1 h, centrifuged at 4°C (4000 r/min, 30 min), and the serum was aspirated in a 2 mL centrifuge tube, stored in a refrigerator (−80°C). Enzyme-linked immunosorbent assay (ELISA) was used to determine leptin, glucose (Glu), triglyceride (Tg), total cholesterol (Tc), short-chain fatty acids (SCFAs), lipopolysaccharide binding protein (LBP), fasting inducible adipocyte factor (FIAF) and tumor necrosis factor-α (TNF-α). The test kits were leptin Assay Kit (JM-11498M1), Glu Assay Kit (S0104F-1), Tg Assay Kit (S0104F-1) Tg Assay Kit (S0140O-1), Tc Assay Kit (S05042-1), SCFAs Assay Kit (JM-11498M1), LBP Assay Kit (JM-12488M1), FIAF Assay Kit (JM-12613M1), TNF-α Assay Kit (JM-02415M1).

Animals were carefully separated from the liver and interscapular brown adipose tissue (BAT) after execution, the connective tissue and white adipose tissue (WAT) were removed, weighed (accurate to 0.01 g) and placed in 5 mL and 2 mL centrifuge tubes, respectively, placed in liquid nitrogen, then stored in a refrigerator (−80°C). The activity of uncoupling protein 1 (UCP1) was determined by ELISA Assay Kit (JM-12185M1).

### 2.4. Determination of body composition

Heart, lungs, spleen and kidneys were carefully separated and the attached connective tissue and fat were removed. Blood was blotted from the surface of the organs with filter paper and weighed (accurate to 0.01 g). Digestive tract was removed and the stomach, small intestine, large intestine and cecum were isolated separately, the mesentery and connective tissue and fat of each organ were carefully removed and weighed.

### 2.5. Determination of gut microbiota

#### 2.5.1. DNA extraction

Take 0.1 g of rectal feces and total DNA enriched on the filter membrane was extracted using a centrifugal column-type soil genome extraction kit (DNeasy PowerSoil Kit, Germany).

#### 2.5.2. High throughput sequencing

The concentration of the purified PCR product was determined using a Nanodrop 2000 spectrophotometer, with a nucleic acid concentration higher than 10 ng/uL and purity (A260/A180) greater than 1.8 as valid samples. The purified DNA samples were mixed in equal molarity and sequenced using the Illumina Miseq platform (Illumina, San Diego, CA, USA).

### 2.6. Bioinformatics analysis

The 2 × 250 bp double end sequences were obtained by sequencing on the Illumina Miseq platform (Illumina, San Diego, CA, USA) and these raw data were processed and analyzed using the QIIME platform (version 1.8). The double-end sequences were first spliced using Flash software (version 1.2.111) and then matched to a unique barcode label for each sample. Low quality sequences (sequence length less than 300 or base mass fraction less than 30) were removed during the splicing process. Chimeras were removed from the sequences using Use arch 7.0 software, and all sequences were subsequently clustered into operational taxonomic units (OTUs) with 97% similarity by the Uclust algorithm. The sequence with the longest sequence length was selected as the representative sequence and the Ribosomal Database Project database was used to annotate the sequence classification information. Finally, the sequences of all samples were normalized using the “Daisy chopper” script code, with a standard of 5437 sequences per sample.

### 2.7. Data analysis

#### 2.7.1. Microbial community composition

A percentage stacked bar chart was created using origin 2018 to describe the bacterial community.

#### 2.7.2. α and β diversity

α diversity was estimated by 2 diversity indicators: chao1 and shannon diversity and described by creating box plots using Origin 2018, and Kruskal-Wallis *H*-test was used in SPSS 21 to analysis if differences in diversity between the two groups. The reason we chose the non-parametric test here was that these two indicators did not conform to the homogeneity of variance. β diversity: community structure was described using QIIME and Origin 2018. Based on unweighted and weighted UniFrac distance matrices, PERMANOVA (Permutational multivariate analysis of variance) was used to calculate the difference among the groups. The unweighted UniFrac distance depends on phylogenetic relationships and OTU species abundance, while species absence/presence and phylogenetic relationships are considered by the weighted UniFrac distance. Then principal co-ordinates analysis (PCoA) was used to visualize the β diversity of all samples.

#### 2.7.3. Venn diagram

Common and unique parameters between groups were analyzed via Venn diagrams implemented online in Venn 2.1.^1^

#### 2.7.4. Enrichment analysis

We used one-way of variance analysis to compare the distribution differences of microbial abundance among groups, and the variable was different treatment group. The genera of microorganisms with significant differences in distribution between groups were screened out, and the heatmap was drawn by R packages “vegan,” “permute,” and “gplots” for visualization. The prefixes “o” and “f” represented the order and family level of unidentified genera, respectively.

#### 2.7.5. Heat map of the correlation between environmental physicochemical properties and dominant microorganisms in the feces of *E. miletus* in different regions

Pearson analysis using SPSS 21 and R3.6.2 were used to obtain the correlation heat map.

#### 2.7.6. RDA analysis

Redundancy analysis (RDA) was used to assess the correlation between dominant genera (top 9) and physicochemical factors using Canoco 5.0.

#### 2.7.7. Network analysis

Use R3.6.2 and Gephiv.0.9.2 software to further analyze these results to generate network analysis (*P* < 0.05, | r | > 0.4). R was used to calculate the correlation between these microorganisms, and the network analysis diagram was drawn based on the correlation matrix. With the help of Gephi, we further calculated the topological characteristics (namely modularity) of the network.

#### 2.7.8. Physiological indicators analysis

The data was analyzed using SPSS 26.0 software, and the differences in various indicators between the two regions were analyzed using two-way ANOVA or two-way ANCOVA (Region × Diet), with body mass as the covariate. The results are expressed as mean ± SE, with *P* < 0.05 indicating significant differences.

## 3. Results

The current study collected 41 samples to extract DNA and amplify PCR products. After removing low-quality sequences, chimeras, monomers, and chloroplasts, each sample was standardized to 3147 sequences.

### 3.1. Microbial community composition

**FIGURE 1:**
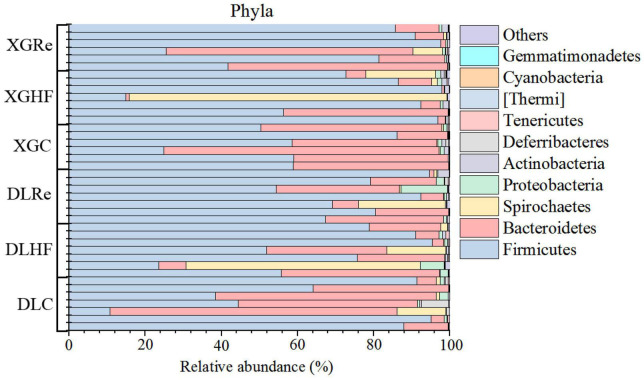
Microbial phylum horizontal community composition in *Eothenomys miletus.*

**FIGURE 2:**
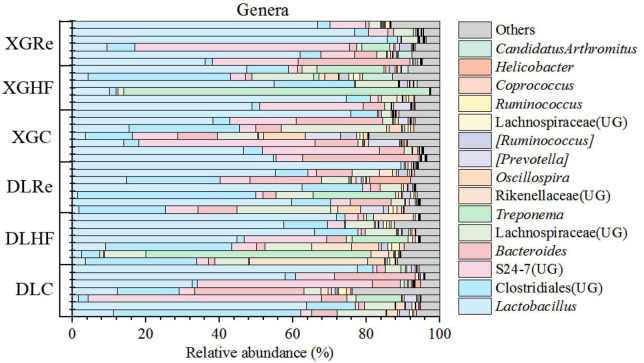
Microbial compositions at the genus level in *Eothenomys miletus.*

The dominant phyla of fecal microorganisms of *E. miletus* in two regions were Firmicutes, Bacteroidetes and Spirochaetes, and the average relative abundance in all groups were 67.99, 24.00, and 5.78%, respectively (Figure 1). The dominant genera of fecal microorganisms were *Lactobacillus*, Clostridiales (UG) and S24-7 (UG), and the average relative abundance in all groups were 40.51, 12.44, and 11.72%, respectively (Figure 2).

### 3.2. α and β diversity analysis

The correlation between the physiological indicators in DL and the dominant genera of *E. miletus* feces (the top ten relative abundance of all samples) was shown in Figure 9A.

**FIGURE 3:**
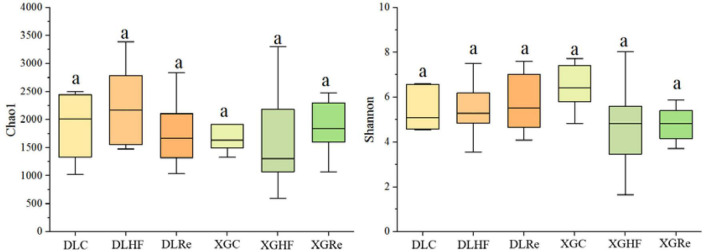
Alpha diversity of fecal microorganisms of *Eothenomys miletus.*

**FIGURE 4:**
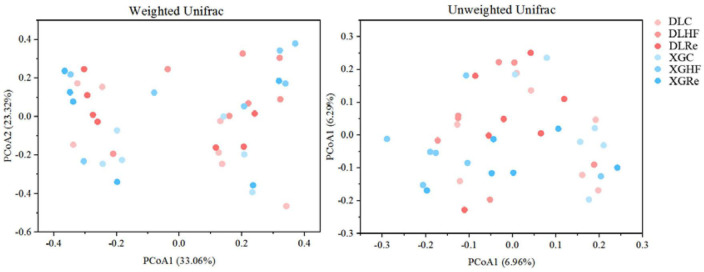
Beta diversity of fecal microorganisms of *Eothenomys miletus.*

Chao1 and Shannon diversity of fecal microorganisms in *E. miletus* was shown in Figure 3. According to the results of PERMANOVAs, there was an overall difference in the beta-diversity (based on unweighted matrix) between groups, which was mainly contributed by the significant differences (*P* < 0.05) between XGC and XGHF groups, as well as between XGRe and DLRe groups (Figure 4 and Table 3).

**TABLE 3:** Permutational multivariate analysis of variance (PERMANOVA) test of fecal microorganisms of *Eothenomys miletus.*

PERMANOVA	Unweighted UniFrac			Weighted UniFrac		
	***F***	***R* ^2^**	***P***	***F***	***R* ^2^**	***P***
DLC vs. DLHF	0.934	0.072	0.453	1.561	0.115	0.182
DLHF vs. DLRe	1.254	0.095	0.214	2.379	0.165	0.098
XGC vs. XGHF	3.268	0.229	0.010*	1.867	0.145	0.142
XGHF vs. XGRe	1.439	0.116	0.147	0.881	0.074	0.426
DLC vs. XGC	0.961	0.080	0.432	0.513	0.045	0.701
DLHF vs. XGHF	1.210	0.092	0.275	0.432	0.035	0.793
DLRe vs. XGRe	1.810	0.141	0.041*	1.441	0.116	0.206

*P < 0.05 indicates significant difference.

### 3.3. Distribution of common and unique microorganisms

**FIGURE 5:**
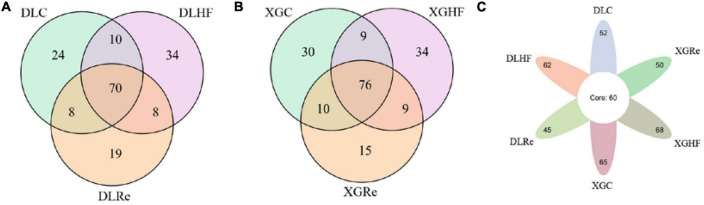
Venn diagram of fecal microorganisms of *Eothenomys miletus*. **(A)** Comparison among different groups in DL. **(B)** Comparison among different groups in XGLL. **(C)** Comparison among different groups between DL and XGLL.

There were 70 genera of fecal microorganisms of *E. miletus* in DL. Among them, there were 24 genera unique to the DLC group, 34 genera unique to the DLHF group, and 19 genera unique to the DLRe group (Figure 5A). There were 76 genera of fecal microorganisms of *E. miletus* in XGLL. There were 30 genera unique to the XGC group, 34 genera unique to the XGHF group, and 15 genera unique to the XGRe group (Figure 5B). There were 71 genera of fecal microorganisms of *E. miletus* in different regions and periods. Among them, there were 52 genera unique to the DLC group, 62 genera unique to the DLHF group, and 45 genera unique to the DLRe group, 65 genera in the XGC group, 68 genera in the XGHF group, and 50 genera in the XGRe group (Figure 5C).

### 3.4. Analysis of microbial enrichment differences

**FIGURE 6:**
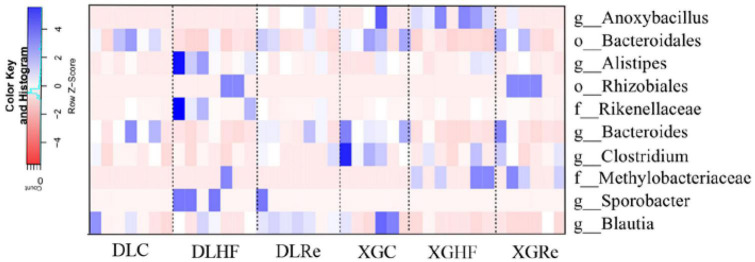
Microbiological analyses of the feces of *Eothenomys miletus.*

The relative abundance of microorganisms in the feces of *E. miletus* in DL and XGLL were different (Figure 6). In DL, Bacteroidales, *Bacteroides*, and *Blautia* were enriched in the DLC group (*P* < 0.05). Compared to the DLC and DLRe groups, *Alistipes*, *Sporobacter*, and Rikenellaceae were enriched in the DLHF group (*P* < 0.05), while *Blautia* was significantly enriched in DLRe group (*P* < 0.05). The distribution of enriched microorganisms in XGLL was significantly different from that in DL. Compared to the XGHF and XGRe groups, *Bacteroides* and *Clostridium* genera were significantly enriched in XGC group (*P* < 0.05), *Anoxybacillus* and *Methylobacteriaceae* were significantly enriched in XGHF group (*P* < 0.05), and Bacteroidales, Rhizobiales, and *Methylobacteriaceae* were significantly enriched in XGRe group (*P* < 0.05) (Figure 6).

### 3.5. Effects of high fiber foods on physiological indicators

**FIGURE 7:**
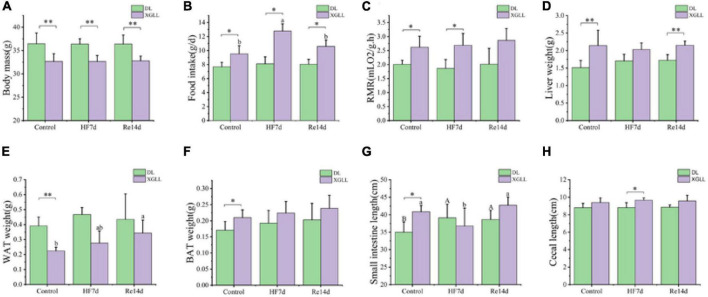
Effects of region and high-fiber food on morphological indicators of *Eothenomys miletus*. **(A)**: Body mass, **(B)**: food intake, **(C)** RMR, **(D)**: liver weight, **(E)**: small intestine length, **(F)**: cecum length, **(G)**: WAT weight, **(H)**: BAT weight. Data are mean ± SE. Different letters denote significant differences among treatments in the same region, capital letters refer to the DL region, and lowercase letters refer to the XGLL region. *P < 0.05, **P < 0.01, DL vs. XGLL on the same day.

Body mass of *E. miletus* was significantly affected by the region (*F* = 52.105, *P* < 0.001), which in XGLL was significantly lower than that of DL. Food intake were significant influenced by region and diet (Region: *F* = 47.894, *P* < 0.001; Diet: *F* = 8.609, *P* < 0.001; Region × Diet: *F* = 8.609, *P* < 0.001). Food intake in DL was lower than that in XGLL, and the food intake in the XGHF group was higher than that in the XGC group and XGRe group. The effect of region on the RMR was significant (*F* = 18.656, *P* < 0.001). RMR of the XGC and XGHF group were higher than that of the DLC and DLHF group. Liver mass of *E. miletus* in the XG group was significantly higher than that in the DL group (*F* = 24.645, *P* < 0.001). The lung mass and small intestine length of *E. miletus* were significantly affected by the interaction of region and diet (Lung weight: Region × Diet: *F* = 3.729, *P* = 0.034; Small intestine length: Region × Diet: *F* = 5.781, *P* = 0.007). Small intestine length of the DLC group was lower than that of other groups, which was the longest in the XGHF group. Region had a significant effect on the cecum length (*F* = 7.49, *P* = 0.01), and the XG group was higher than the DL group. WAT mass of *E. miletus* was significantly affected by diet (*F* = 3.336, *P* = 0.048). In the control group, WAT weight of the DLC group was higher than that of the XGC group, and the WAT weight of the XGRe group was higher than that of the control group. Region also had a significant effect on the BAT weight of *E. miletus* (*F* = 4.958, *P* = 0.033). BAT weight of the XGC group was higher than that of the DLC group (Figure 7). The influence of region and diet on other indexes of *E. miletus* were not significant (Table 4).

**TABLE 4:** Effects of region and high-fiber food on physiological indicators in *Eothenomys miletus.*

Para- meter	DL			XGLL			Region		Diet		Region × Diet	
	**Con group *n* = 7**	**HF group *n* = 7**	**Re group *n* = 7**	**Con group *n* = 7**	**HF group *n* = 7**	**Re group *n* = 6**	***F***	***P***	***F***	***P***	***F***	***P***
Body mass (g)	36.441 ± 2.318	36.384 ± 1.152	36.391 ± 1.919	32.670 ± 1.652	32.661 ± 1.289	32.790 ± 1.023	52.105	<0.001	0.006	0.994	0.01	0.991
Food intake (g/d)	7.677 ± 0.638	8.110 ± 0.984	8.043 ± 0.702	9.539 ± 1.150	12.781 ± 1.037	10.600 ± 0.900	47.894	<0.001	13.872	<0.001	8.609	<0.001
RMR (mLO_2_/g.h)	2.009 ± 0.143	1.864 ± 0.313	2.014 ± 0.563	2.622 ± 0.389	2.687 ± 0.422	2.864 ± 0.422	18.656	<0.001	0.683	0.512	0.406	0.669
Heart weight (g)	0.252 ± 0.073	0.242 ± 0.063	0.270 ± 0.033	0.252 ± 0.024	0.272 ± 0.029	0.254 ± 0.075	2.767	0.105	0.111	0.895	0.719	0.495
Liver weight (g)	1.512 ± 0.203	1.703 ± 0.189	1.721 ± 0.162	2.142 ± 0.438	2.032 ± 0.184	2.151 ± 0.120	24.645	<0.001	0.669	0.519	1.399	0.261
Spleen weight (g)	0.089 ± 0.195	0.109 ± 0.027	0.099 ± 0.307	0.104 ± 0.318	0.100 ± 0.015	0.099 ± 0.011	0.36	0.552	0.396	0.676	0.905	0.414
Kidney weight (g)	0.528 ± 0.071	0.489 ± 0.065	0.478 ± 0.090	0.511 ± 0.083	0.586 ± 0.071	0.594 ± 0.063	1.557	0.221	0.24	0.788	3.181	0.054
Lung weight (g)	0.266 ± 0.046	0.274 ± 0.043	0.252 ± 0.038	0.239 ± 0.361	0.254 ± 0.049	0.303 ± 0.017	0.016	0.807	1.279	0.291	3.729	0.034
Small intestine length (cm)	35.000 ± 2.930	39.143 ± 3.868	38.643 ± 2.607	40.871 ± 1.745	36.800 ± 5.128	42.733 ± 2.320	1.371	0.25	2.956	0.066	5.781	0.007
Cecal length (cm)	8.814 ± 0.488	8.819 ± 0.562	8.857 ± 0.264	9.396 ± 0.532	9.657 ± 0.270	9.575 ± 0.649	7.49	0.01	0.301	0.742	0.249	0.781
WAT weight (g)	0.392 ± 0.059	0.468 ± 0.046	0.435 ± 0.170	0.224 ± 0.024	0.276 ± 0.082	0.344 ± 0.086	3.357	0.076	3.336	0.048	1.186	0.318
BAT weight (g)	0.171 ± 0.027	0.193 ± 0.039	0.203 ± 0.051	0.210 ± 0.024	0.224 ± 0.036	0.239 ± 0.040	4.958	0.033	2.314	0.114	0.042	0.958

Data are mean ± SE. The differences in various indicators between the two regions were analyzed using two-way ANOVA or two-way ANCOVA, with body mass as the covariate.

**FIGURE 8:**
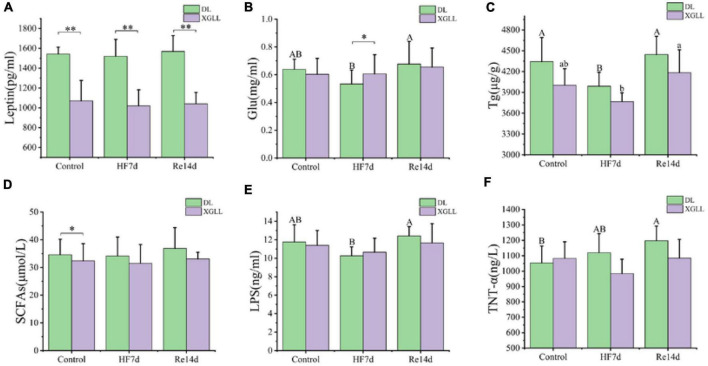
Effects of region and high-fiber food on physiological indices of *Eothenomys miletus.* **(A)**: Leptin, **(B)**: Glu, **(C)**: Tg, **(D)**: SCFAs, **(E)**: LBP, **(F)**: TNT-α. Data are mean ± SE. Different letters denote significant differences among treatments in the same region, capital letters refer to the DL region, and lowercase letters refer to the XGLL region. *P < 0.05, **P < 0.01, DL vs. XGLL on the same day.

Leptin and UCP1 of *E. miletus* were significantly affected by region (Leptin: *F* = 49.832, *P* < 0.001; UCP1: *F* = 7.756, *P* = 0.009). Leptin in DL was higher than that of XGLL, and UCP1 in XGLL was higher than that of DL. The influence of the region on the SCFAs was significant (*F* = 4.996, *P* = 0.032). The SCFAs of the DLC and XGC group were significantly different, and the difference in DL was higher than that in XGLL. Diet had a significant effect on LBP in *E. miletus* [*F*_(3.633)_, *P* = 0.037]. LBP in DLRe group was higher than that in DLHF group (Figure 8). The influence of region and diet on other indexes of *E. miletus* were not significant (Table 5).

**TABLE 5:** Effects of region and high-fiber food on serum physiological indicators in *Eothenomys miletus.*

Parameter	DL			XGLL			Region		Diet		Region × Diet	
	**Con group *n* = 7**	**HF group *n* = 7**	**Re group *n* = 7**	**Con group *n* = 7**	**HF group n = 7**	**Re group *n* = 6**	***F***	***P***	***F***	***P***	***F***	***P***
Tg (μg/g)	4344.103 ± 47.236	3990.627 ± 199.447	4446.074 ± 261.753	4003.2743 ± 238.226	3768.300 ± 126.118	4184.755 ± 329.208	11.487	0.002	10.002	<0.001	0.190	0.828
Tc (μg/g)	1838.876 ± 264.911	1869.644 ± 221.468	1786.769 ± 252.200	1701.406 ± 290.353	1727.321 ± 357.620	1733.683 ± 252.851	0.119	0.732	0.083	0.921	0.097	0.907
Glu (mg/ml)	0.639 ± 0.073	0.534 ± 0.099	0.677 ± 0.163	0.603 ± 0.115	0.606 ± 0.139	0.655 ± 0.137	0.294	0.591	2.027	0.147	0.757	0.477
Leptin (pg/ml)	1542.616 ± 68.808	1519.344 ± 171.092	1568.811 ± 159.398	1069.974 ± 206.603	1020.632 ± 160.576	1040.873 ± 113.865	49.832	<0.001	0.250	0.780	0.099	0.906
UCP1 (pg/ml)	1298.614 ± 290.171	1277.614 ± 214.712	1264.221 ± 314.209	1433.350 ± 234.681	1477.343 ± 163.719	1495.917 ± 90.224	7.756	0.009	0.014	0.986	0.140	0.870
SCFAss (μmol/L)	34.600 ± 5.595	34.184 ± 6.788	36.910 ± 7.492	32.396 ± 6.223	31.527 ± 6.764	33.130 ± 2.355	4.996	0.032	0.480	0.623	0.048	0.954
FAIF (ng/L)	57.377 ± 11.125	54.710 ± 7.498	55.154 ± 8.742	58.894 ± 10.596	55.714 ± 14.516	58.312 ± 5.141	0.165	0.687	0.297	0.745	0.047	0.954
LBP (ng/ml)	11.769 ± 1.858	10.276 ± 0.955	12.413 ± 1.004	11.404 ± 1.596	10.659 ± 1.516	11.658 ± 2.088	0.001	0.977	3.633	0.037	0.472	0.628
TNT-α (ng/L)	1053.574 ± 109.626	1120.210 ± 122.758	1197.591 ± 95.090	1083.013 ± 107.601	984.707 ± 92.828	1085.007 ± 120.964	2.179	0.149	2.475	0.099	2.289	0.117

Data are mean ± SE. The differences in various indicators between the two regions were analyzed using two-way ANCOVA, with body mass as the covariate.

### 3.6. Relationship between physiological indicators and microorganisms in *E. miletus*

**FIGURE 9:**
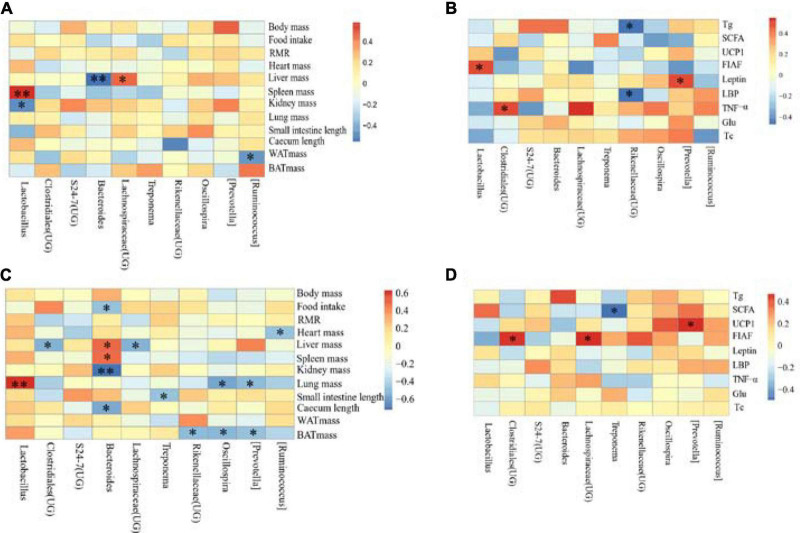
Correlation heat map between physiological indicators and dominant microorganisms of *Eothenomys miletus*. *P < 0.05, **P < 0.01, Pearson correlation analysis were used by SPSS 21. **(A)**: Physiological indicators in DL, **(B)**: serum physiological indicators in DL, **(C)**: physiological indicators in XGLL, **(D)**: serum physiological indicators in XGLL.

The correlation between the physiological indicators in DL and the dominant genera of *E. miletus* feces (the top ten relative abundance of all samples) was shown in Figure 9A. From the graph, it can be seen that liver weight is significantly negatively correlated with the relative abundance of *Bacteroides* (*P* < 0.01), and positively correlated with the relative abundance of Lachnospiraceae (UG) (*P* < 0.05); the relative abundance of Lactobacillus was positively correlated with spleen weight (*P* < 0.01) and negatively correlated with kidney weight (*P* < 0.05). Moreover, there was a significant negative correlation between WAT weight and relative abundance of *Ruminococcus* (*P* < 0.05).

The correlation between the detection indicators in DL and the dominant genera of *E. miletus* feces (the top ten relative abundance of all samples) was shown in Figure 9B. From the graph, it can be seen that there is a significant negative correlation between Tg and LBP and the relative abundance of Rikenellaceae (UG) (*P* < 0.05); There is a positive correlation between the relative abundance of leptin and *Prevotella* (*P* < 0.05); FIAF is positively correlated with the relative abundance of *Lactobacillus* (*P* < 0.05); There was a significant positive correlation between the relative abundance of TNF- α and Clostridiales (UG) (*P* < 0.05).

The correlation between the physiological indicators in XGLL and the dominant genera of *E. miletus* feces (the top ten relative abundance of all samples) was shown in Figure 9C. From the graph, it can be seen that there is a significant negative correlation (*P* < 0.05) between the relative abundance of BAT mass and *Prevotella*, *Oscillospira*, and Rikenellaceae (UG); The relative abundance of *Bacteroides* was negatively correlated with food intake, kidney weight, and cecal length (*P* < 0.05), while spleen weight was positively correlated (*P* < 0.05); The liver weight is positively correlated with the relative abundance of *Bacteroides*, but negatively correlated with the relative abundance of Clostridiales (UG) and Lachnospiraceae (UG) (*P* < 0.05); The length of the small intestine is negatively correlated with the relative abundance of Treponema (*P* < 0.05); The relative abundance of *Ruminococcus* was significantly negatively correlated with heart weight (*P* < 0.05); The lung weight was significantly positively correlated with the relative abundance of *Lactobacillus* (*P* < 0.01), but negatively correlated with the relative abundance of *Prevotella* and *Oscillospira* (*P* < 0.05).

The correlation between the detection indicators in XGLL and the dominant genera of *E. miletus* feces (the top ten relative abundance of all samples) was shown in Figure 9D. From the graph, it can be seen that the relative abundance of Lachnospiraceae (UG) and Clostridiales (UG) is significantly positively correlated with FIAF (*P* < 0.05); UCP1 was significantly positively correlated with the relative abundance of *Prevotella* (*P* < 0.05); There was a significant negative correlation between the relative abundance of SCFAs and *Treponema* (*P* < 0.05).

**FIGURE 10:**
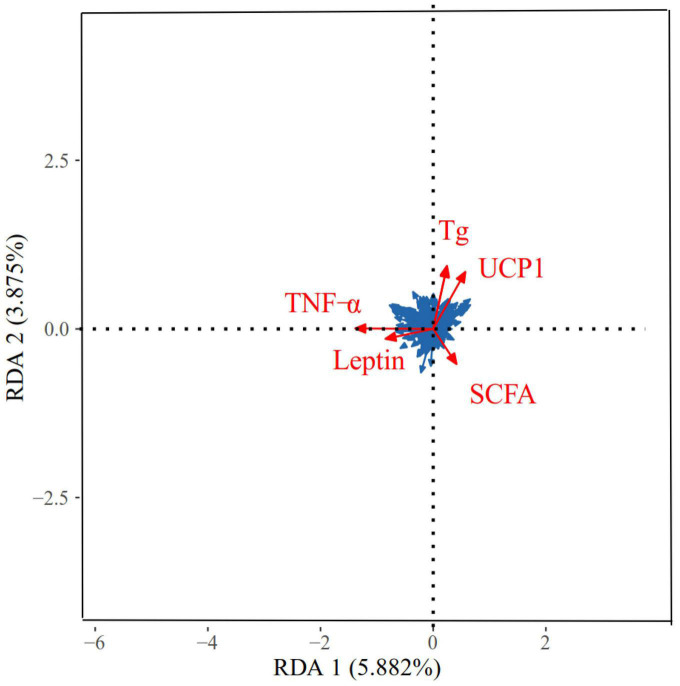
Redundancy analyses (RDA) of the correlation between physiological indicators and dominant microbial communities in *Eothenomys miletus*. RDA was used to assess the correlation between dominant genera (top 9) and physicochemical factors using Canoco 5.0.

The correlation between the physiological indicators in different regions and the dominant OTU in the feces of *E. miletus* is shown in Figure 10. The physiological indicators that have a major impact on the microbial community were TNF-α, Tg, UCP1, SCFAs, and leptin. The RDA results showed that the explanatory power of these physiological indicators on microorganisms with main axes were 5.88 and 3.88%, respectively.

### 3.7. Co-occurrence network of fecal microorganisms of *E. miletus* in different regions

**FIGURE 11:**
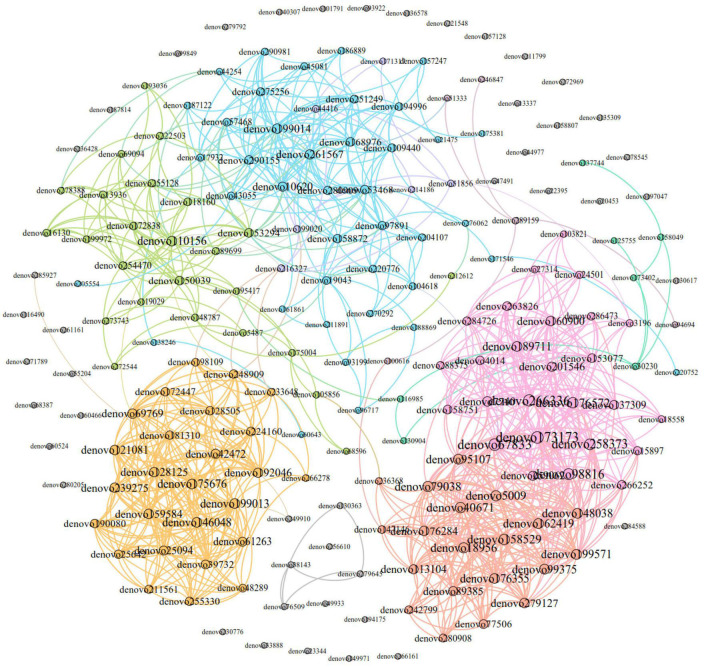
Dominant OTU co-occurrence network of *Eothenomys miletus* between two regions. Nodes were colored according to their modular characteristics, and the same color represented a close correlation within the module. The colors of the edges were consistent with their corresponding node. The size of a node was positively correlated with the degree, and the larger it was, the more connections it had in the network.

The dominant OTUs correlation network of *E. miletus* feces in different regions was shown in Figure 11. The network analysis included the top 200 OTUs with relative abundance, and based on Gephi 0.9.2, a network with 200 nodes and 1149 edges was constructed, including 1139 positive edges and 10 negative edges. It means that the microorganisms in the advantage OTU co-occurrence network were mainly cooperative.

## 4. Discussion

### 4.1. Intestinal microorganisms of *E. miletus*

Animal species are abundant and their feeding habits are complex, and these complex ecological characteristics also shape the different gut microbiota characteristics. Previous researches results have shown that gut microbiota in *E. miletus* were highly adapted to their herbivorous habits (Yan and Zhu, 2023). In the present study, the main dominant organisms in the gut microflora of *E. miletus* at the facultative level were Firmicutes, Bacteroidetes and Spirochaetes, and a high ratio of F/B is essential for the digestion of cellulose and hemi fibre in phytophagous animals, and also in the gut microflora of small mammals, such as *Ochotona curzoniae*, *Meriones unguiculatus*, where the ratio of F/B were also high (Ley et al., 2006; Steelman et al., 2012; Li et al., 2016; Khakisahneh et al., 2020). The HF group possessed higher F/B ratios compared to previous experiments and the control group in this experiment, and Spirochaetes were found to be associated with Xylan and Carboxymethyl cellulose fermentation (Flint et al., 2008). The dominant organisms in *E. miletus* gut microbiota at the genus level were the same as in previous studies, *Lactobacillus*, Clostridiales (UG) and S24-7 (UG), but with different proportions. *Lactobacillus* had the highest percentage of 40.51% in this experiment and S24-7 (UG) had the highest percentage of 29.32% in the previous experiment. It has been shown that *Lactobacillus*, Clostridiales (UG) and S24-7 (UG) were associated with the digestive degradation of cellulose, and *Lactobacillus* also assists the host in releasing phytochemicals with potent antioxidant and anti-inflammatory activities from ingested fiber, and from the results of the present experiments, it is hypothesized that *Lactobacillus* may possess a higher level of fitness for high-fiber foods (Van Dyke and McCarthy, 2002; Russell and Duthie, 2011; Geirnaert et al., 2012; Ormerod et al., 2016; Serena et al., 2018). The results of the present experiment indicated that the high proportion of F/B and the enrichment of cellulose degrading bacteria in the intestinal microbiota of *E. miletus* enhanced their adaptability to high-fiber foods.

### 4.2. Effects of high-fiber food on intestinal microorganisms and physiological indicators of *E. miletus*

Body mass directly reflects the energy balance in mammals (Zhu et al., 2010). In the present study, high fiber food had no significant effect on body mass of *E. miletus*, but it had a significant effect on food intake, which was higher in the HF group than in the other groups. For high-fiber food, which was difficult to digest to obtain nutrients, *E. miletus* may regulate their body weight homeostasis by increasing the amount of food intake so as to obtain more energy. Compensating for decreased digestibility by increasing food intake is a common strategy in small mammals, such as the herbivorous *Dicrostonyx groenlandicus* and *Meriones unguiculatus*, and has also been adopted by *E. miletus* for high-fiber foods (Nagy and Negus, 1993; Zhao and Wang, 2007). Following a high-fiber diet, small intestine grew in length, and the mass of the digestive tract (except the stomach) also increased, *E. miletus* showed similar responding to high-fiber foods (Kass et al., 1980).

Host food resources are the main factor affecting gut microbiota diversity, and a high-fiber diet can increase gut microbiota diversity (Hu et al., 2018). According to the results of PERMANOVA, it was found that the differences in β-diversity between XGC group and XGHF group, and between XGRe and DLRe groups were significant, indicating that there were structural differences in the gut microorganisms. Venn diagram results showed that HF group contained the most species of gut microbiota, followed by the control group, and the Re group had the smallest number. The high-fiber food might increase the β-diversity of intestinal microorganisms in *E. miletus*.

The gut microbiota can assist the host in digesting difficultly decomposed food, providing nutrients that were originally unavailable, and allowing the host to flexibly respond to constantly changing environments (Salyers et al., 1977; Lindsay et al., 2020). In the present study, through the enrichment of intestinal microorganisms in each group of *E. miletus*, it was found that Bacteroidales and *Bacteroides* were enriched among DLC, XGC and XGRe groups. Studies have shown that *Bacteroides* could supplement eukaryotic genome by degrading enzymes targeting resistant dietary polymers, and it is believed that Bacteroidota had the higher fiber degradation potential compared with other phyla (Martínez et al., 2010; El Kaoutari et al., 2013). In addition to typical cellulose degrading bacteria, some probiotics capable of digesting cellulose were enriched in the intestinal microorganisms of *E. miletus*. Between the DLC group and the DLRe group, *Blautia* was enriched, which can prevent pathogen colonization by producing bacteriocin, and exhibited anti-inflammatory properties and maintained glucose homeostasis by up-regulating the production of regulatory T cells and SCFAs (Liu et al., 2021). *Alistipes*, *Sporobacter* and Rikenellaceae enriched in the DLHF group were all probiotic bacteria, and studies have shown that *Alistipes*, Rikenellaceae, and *Sporobacter* all produced acetic acid and were involved in the uptake and metabolism of SCFAs, and Rikenellaceae could degraded aromatic compounds and produced SCFAs, SCFAs play an important role in appetite regulation, energy metabolism, inflammation and disease as end products of fermentation of dietary fiber and resistant starch, moreover, Rikenellaceae were considered to be a key bacterium for the control of intestinal infections or inflammation in the treatment of non-infectious colitis (Morrison and Preston, 2016; Parker et al., 2020; Qiu et al., 2022; Nishihara et al., 2023). *Anoxybacillus* was enriched in the XGHF group, and research suggested that it was beneficial for the proliferation of probiotics such as fecal bacteria and *Lactobacillus* rosenbergii (Liu et al., 2011). Current research suggested that high-fiber foods as prebiotics can promote the growth of specific members of the resident gut microbiota, stimulated the production of SCFAs, lower pH, and maintain intestinal homeostasis (Scott et al., 2013). However, it is worth noting that *Methylobacteriaceae*, which was enriched in the XGHF and XGRe group, was an opportunistic pathogen that poses a threat to human health and has been found to be able to enter the silkworm gut through food (Consiglieri et al., 2020; Li et al., 2020). In our study, the abundance of dominant fiber degrading bacteria increased after the high-fiber diet in *E. miletus*, thus digesting more fiber to provide energy for them. At the same time, it promoted the proliferation of probiotics, participated in the regulation of immunity and energy metabolism of *E. miletus* through SCFAs and other metabolites, and maintained the energy balance and health of *E. miletus*.

Through redundancy analysis, it was found that SCFAs, leptin, UCP1, and Tg may have a major impact on the microbial community. Studies have found that SCFAs not only play an important role in maintaining energy balance and increasing energy regulation such as insulin, but also promote intestinal barrier function and intestinal immune homeostasis through various mechanisms, thereby regulating the affinity of fibers in the intestine through immune regulation and increasing the proportion of beneficial bacteria in the gut microbiota (Chawla and Patil, 2010). Propionate and butyrate can participate in the body’s energy metabolism, increase energy consumption, and down regulate TNT-α gene expression and release of inflammatory factors to maintain intestinal homeostasis (Mattace Raso et al., 2013). In the current experiment, there was a significant negative correlation between the WAT weight and the relative abundance of *Ruminococcus*, and between Tg and the relative abundance of Rikenellaceae (UG) in DL; There was a significant negative correlation between BAT mass and the relative abundance of *Prevotella*, Rikenellaceae (UG) and *Oscillospira* in XGLL. Research showed that *Ruminococcus*, Rikenellaceae and *Prevotella* were all fiber degrading bacteria, which can degrade structural carbohydrates and starch, and can produce SCFAs such as acetate and propionate (Bi et al., 2018; Qiu et al., 2022). In this experiment, under the condition of high-fiber food, it is speculated that the metabolites of *E. miletus* after their intestinal microorganisms digested cellulose may participate in the regulation of energy metabolism of *E. miletus* by inhibiting the production of BAT and WAT. Moreover, the relative abundance of LBP was significantly negatively correlated with the relative abundance of Rikenellaceae and positively correlated with the relative abundance of *Lactobacillus* in DL, while the relative abundance of Lachnospiraceae (UG) and Clostridiales (UG) were significantly positively correlated with the relative abundance of *Lactobacillus* in XGLL. Research has shown that Lachnospiraceae utilizes lactic acid and acetic acid to produce butyric acid; *Lactobacillus* can improve intestinal integrity, reduce systemic LBP level, regulate lipid metabolism, and regulate the composition of gut microbiota and SCFAs (Lim et al., 2016; Mollica et al., 2017). In our experiment, it showed that *E. miletus* can regulate body immunity by inhibiting LBP by specific flora and promoting the production of LBP inhibitory factor FIAF, so as to maintain body health.

Liver is one of the thermogenic organs of small mammals (Zhu et al., 2011). In the present study, after high-fiber diet acclimation, the liver weight of DL increased, while that of XGLL decreased, and increased after refeeding. Analyses of gut microorganisms on related indicators revealed that liver weight of DL was significantly negatively correlated with the relative abundance of *Bacteroides* and positively correlated with the relative abundance of Lachnospiraceae (UG), while liver weight of XGLL was positively correlated with the relative abundance of *Bacteroides*, and negatively correlated with the relative abundance of Clostridiales (UG) and Lachnospiraceae (UG). *Prevotella* relative abundance is positively correlated with leptin and UCP1 (Pfannenberg et al., 2010), suggested that under high-fiber food conditions, *Bacteroides*, Lachnospiraceae (UG) may assist in thermogenesis in the liver of *E. miletus* through metabolites, and *Prevotella* metabolites may be involved in the regulation of thermogenesis by modulating the contents of leptin and UCP1, and thus enhancing its adaptation to the high-fiber food environment.

Specific flora had a preference for certain specific food components in different environments, and the metabolites of the flora would participate in the regulation of the intestinal microenvironment and host homeostasis, and the intestinal microenvironment will be more conducive to the reproduction and growth of the relevant flora in large quantities and colonization, so as to maintain the balance of the host homeostasis (Moeller and Sanders, 2020). In the present study, under the condition of high-fiber food, different bacterial groups participated in food digestion or energy metabolism etc., of *E. miletus*. The co-occurrence network results showed that intestinal microorganisms were dominated by cooperation, and positive cooperation played an important role in adaptation to high-fiber food (Abbas et al., 2020).

### 4.3. Regional differences in body mass regulation under high-fiber food

Under the condition of high-fiber diet, body mass, food intake, RMR, liver weight, cecum length, BAT weight, leptin, UCP1 and SCFAs of *E. miletus* were significantly different between DL group and XGLL group. The elevation of XGLL was higher than that of DL, and the winter temperature and food stress were greater in XGLL. The higher metabolism in XGLL may related to heavier liver and BAT mass, and higher UCP1 content was its adaptation to low temperature environment and longer cecum is an adaptation to the high-fiber food, which was similar to our previous results (Scott et al., 2013). However, it is worth noting that the DL *E. miletus* decreased its RMR while increased its intake of high-fiber food, whereas the XGLL *E. miletus* adopted higher food intake and RMR. It is assumed that the DL *E. miletus* was not able to digest the high-fiber food fast enough to obtain energy, and instead, it adopted a lower metabolism to maintain its energy balance.

The results of this experiment showed that the composition of intestinal microorganisms in the XGLL group was more than that in the DL group. After the high-fiber diet, the difference between the two regions was reduced, and there was structural difference in intestinal microorganisms in the refeeding groups. It showed that the food diversity of the XGLL was higher than that of Dali, and the high-fiber food increased the diversity of intestinal microorganisms, indicating that food resources were poorer and the food contains higher levels of indigestible cellulose in the winter of XGLL, and that gut microorganisms assist the XGLL *E. miletus* in facing the stress of winter food resources by altering their structure and diversity (Varel and Dehority, 1989). The intestinal microorganisms in DL and XGLL were also different in control groups. In addition to the dominant bacteria for cellulose degradation, more probiotics appeared in DL after the high-fiber diet, while pathogenic bacteria appeared in XGLL. Therefore, for the herbivorous *E. miletus*, high-fiber food may also enrich pathogenic bacteria in addition to reducing the digestibility and increasing the stress for *E. miletus* in winter.

In conclusion, the present study for the first time explored the effects of high-fiber food on the intestinal microorganisms of *E. miletus* at different altitudes. It was found that high-fiber food affected the diversity and enrichment of intestinal microorganisms, and there were similarities and differences in the effects of high-fiber food on *E. miletus* at different altitudes. Under the condition of high-fiber food, the intestinal microorganisms assist *E. miletus* to obtain energy from indigestible cellulose through the enrichment of fiber degrading bacteria, produced SCFAs, participated in energy metabolism, immune regulation, etc., in different forms, and also affected the colonization of intestinal microorganisms, so as to maintain the stability of the flora. High-fiber food also promoted the enrichment of probiotics in the intestinal microbiota of *E. miletus*, but pathogenic bacteria also appeared. A diverse and responsive microbial community may be a key strategy for living in extreme climates (Tsuji et al., 2013). Therefore, the plasticity of the composition of the gut microbiota in combination with the large metabolic reservoir of microorganisms of *E. miletus* provided an important adaptation to a high-altitude environment with low winter temperatures and scarce food resources. Moreover, we found that there had relationships between the physiological processes and the changes of relative intestinal bacteria; however, further follow-up research is still needed on these relationships.

## Data availability statement

The datasets presented in this study can be found in online repositories. The names of the repository/repositories and accession number(s) can be found below: EBI—PRJEB61600.

## Ethics statement

All animal procedures were within the rules of Animals Care and Use Committee of School of Life Sciences, Yunnan Normal University. This study was approved by the committee (13-0901-011). The study was conducted in accordance with the local legislation and institutional requirements.

## Author contributions

WeZ: Investigation, Methodology, and Writing—original draft. TJ: Investigation, Methodology, and Writing—original draft. HZ: Methodology, Resources, and Writing—original draft. WaZ: Software, Writing—original draft, and Writing—review and editing.

## Footnotes

1
https://bioinfogp.cnb.csic.es/tools/venny/index.html

## References

[B1] Abbas W. Howard J. T. Paz H. A. Hales K. E. Wells J. E. Kuehn L. A. (2020). Influence of host genetics in shaping the rumen bacterial community in beef cattle. *Sci. Rep.* 10:15101. 10.1038/s41598-020-72011-9 32934296 PMC7493918

[B2] Alberdi A. Aizpurua O. Bohmann K. Zepeda-Mendoza M. L. Gilbert M. T. P. (2016). Do vertebrate gut metagenomes confer rapid ecological adaptation? *Trends Ecol. Evol.* 31 689–699. 10.1016/j.tree.2016.06.008 27453351

[B3] Bi Y. Zeng S. Zhang R. Diao Q. Tu Y. (2018). Effects of dietary energy levels on rumen bacterial community composition in Holstein heifers under the same forage to concentrate ratio condition. *BMC Microbiol.* 18:69. 10.1186/s12866-018-1213-9 29996759 PMC6042446

[B4] Bozinovic F. (1995). Nutritional energetics and digestive responses of an herbivorous rodent (*Octodon degus*) to different levels of dietary fiber. *J. Mammal.* 76 627–637. 10.1016/j.cvex.2009.01.003 19341951

[B5] Chawla R. Patil G. R. (2010). Soluble dietary fiber. *Compr. Rev. Food Sci. Food Safety* 9 178–196.

[B6] Consiglieri E. Xu Q. Z. Zhao K. H. Gärtner W. Losi A. (2020). The first molecular characterization of blue- and red-light photoreceptors from *Methylobacterium radiotolerans*. *Phys. Chem. Chem. Phys.* 22 12434–12446. 10.1039/d0cp02014a 32458860

[B7] Dillon R. J. Vennard C. T. Buckling A. Charnley A. K. (2005). Diversity of locust gut bacteria protects against pathogen invasion. *Ecol. Lett.* 8 1291–1298.

[B8] El Kaoutari A. Armougom F. Gordon J. I. Raoult D. Henrissat B. (2013). The abundance and variety of carbohydrate-active enzymes in the human gut microbiota. *Nat. Rev. Microbiol.* 11 497–504. 10.1038/nrmicro3050 23748339

[B9] Ezenwa V. O. Gerardo N. M. Inouye D. W. Medina M. Xavier J. B. (2012). Animal behavior and the microbiome. *Science* 338 198–199. 10.1126/science.1227412 23066064

[B10] Flint H. J. Bayer E. A. Rincon M. T. Lamed R. White B. A. (2008). Polysaccharide utilization by gut bacteria: Potential for new insights from genomic analysis. *Nat. Rev. Microbiol.* 6 121–131. 10.1038/nrmicro1817 18180751

[B11] Geirnaert A. Debruyne B. Eeckhaut V. Van Immerseel F. Booni N. Van de Wiele T. (2012). In vitro evaluation of the upper gastrointestinal passage of a novel butyrate producing isolate to counterbalance dysbiosis in inflammatory bowel disease. *Commun. Agric. Appl. Biol. Sci.* 77 45–49. 22558754

[B12] Gilbert S. F. Bosch T. C. Ledón-Rettig C. (2015). Eco-Evo-Devo: Developmental symbiosis and developmental plasticity as evolutionary agents. *Nat. Rev. Genet.* 16 611–622. 10.1038/nrg3982 26370902

[B13] Gong X. N. Jia T. Zhang D. Zhu W. L. (2022). Faster response to high-fat diet in body mass regulation from lower altitude population in *Eothenomys miletus* from Hengduan Mountain regions. *Pakist. J. Zool.* 54 167–174. 10.17582/journal.pjz/20200211050216

[B14] Gong Z. D. Wu H. Y. Duan X. D. Feng X. G. Zhang Y. Z. Liu Q. (2001). The species diversity and distribution trends of small mammals in Hengduan Mountains, Yunnan. *Biodivers. Sci.* 9 73–79. 10.3321/j.issn:1005-0094.2001.01.011 30704229

[B15] Han C. Y. Jia T. Yang J. Wang Z. K. Zhu W. L. (2021). Body mass regulation by *Eothenomys miletus* at different areas of the Hengduan Mountains in winter. *Chinese J. Wildlife* 42 86–91. 10.19711/j.cnki.issn2310-1490.2021.01.011

[B16] Hu X. L. Liu G. Li Y. M. Wei Y. T. Lin S. B. Liu S. Q. (2018). High-throughput analysis reveals seasonal variation of the gut microbiota composition within forest musk deer (*Moschus berezovskii*). *Front. Microbiol.* 9:1674. 10.3389/fmicb.2018.01674 30093891 PMC6070636

[B17] Kass M. L. Soest P. J. Pond W. G. Lewis B. A. Mcdowell R. E. (1980). Utilization of dietary fiber from alfalfa by growing swine. I. Apparent digestibility of diet components in specific segments of the gastrointestinal tract. *J. Anim. Sci.* 50 175–191.

[B18] Khakisahneh S. Zhang X. Y. Nouri Z. Wang D. H. (2020). Gut microbiota and host thermoregulation in response to ambient temperature fluctuations. *mSystems* 5 e514–e520. 10.1128/mSystems.00514-20 33082280 PMC7577294

[B19] Kohl K. D. Dearing M. D. (2016). The wood rat gut microbiota as an experimental system for understanding microbial metabolism of dietary toxins. *Front. Microbiol.* 7:1165. 10.3389/fmicb.2016.01165 27516760 PMC4963388

[B20] Kohl K. D. Weiss R. B. Cox J. Dale C. Dearing M. D. (2014). Gut microbes of mammalian herbivores facilitate intake of plant toxins. *Ecol. Lett.* 17 1238–1246. 10.1111/ele.12329 25040855

[B21] Ley R. E. Peterson D. A. Gordon J. I. (2006). Ecological and evolutionary forces shaping microbial diversity in the human intestine. *Cell* 124 837–848. 16497592 10.1016/j.cell.2006.02.017

[B22] Li F. C. Li M. X. Mao T. T. Wang H. Chen J. Lu Z. T. (2020). Effects of phoxim exposure on gut microbial composition in the silkworm, *Bombyx mori*. *Ecotoxicol. Environ. Safety* 189:110011. 10.1016/j.ecoenv.2019.110011 31796255

[B23] Li H. Qu J. Li T. Li J. Lin Q. Li X. (2016). Pika population density is associated with the composition and diversity of gut microbiota. *Front. Microbiol.* 7:758. 10.3389/fmicb.2016.00758 27242770 PMC4870984

[B24] Lim S. M. Jeong J. J. Woo K. H. Han M. J. Kim D. H. (2016). Lactobacillus sakei OK67 ameliorates high-fat diet-induced blood glucose intolerance and obesity in mice by inhibiting gut microbiota lipopolysaccharide production and inducing colon tight junction protein expression. *Nutr. Res.* 36 337–348. 10.1016/j.nutres.2015.12.001 27001279

[B25] Lindsay E. C. Metcalfe N. B. Llewellyn M. S. (2020). The potential role of the gut microbiota in shaping host energetics and metabolic rate. *J. Anim. Ecol.* 89 2415–2426. 10.1111/1365-2656.13327 32858775

[B26] Liu J. Lei Y. Wang F. Yi Y. Liu Y. Wang G. (2011). Immunostimulatory activities of specific bacterial secondary metabolite of Anoxybacillus flavithermus strain SX-4 on carp, Cyprinus carpio. *J. Appl. Microbiol.* 110 1056–1064. 10.1111/j.1365-2672.2011.04963.x 21294820

[B27] Liu X. Mao B. Gu J. Wu J. Cui S. Wang G. (2021). Blautia-a new functional genus with potential probiotic properties? *Gut Microbes* 13 1–21. 10.1080/19490976.2021.1875796 33525961 PMC7872077

[B28] Liu X. Stanford C. B. Yang J. Yao H. Li Y. (2013). Foods eaten by the Sichuan snub-nosed monkey (*Rhinopithecus roxellana*) in Shennongjia National Nature Reserve, China, in relation to nutritional chemistry. *Am. J. Primatol.* 75 860–871. 10.1002/ajp.22149 23589133

[B29] Lou Z. X. Chen W. Gao W. (2000). *Fauna of China.* Beijing: Science Press, 89–95.

[B30] Martínez I. Kim J. Duffy P. R. Schlegel V. L. Walter J. (2010). Resistant starches types 2 and 4 have differential effects on the composition of the fecal microbiota in human subjects. *PLoS One* 5:e15046. 10.1371/journal.pone.0015046 21151493 PMC2993935

[B31] Mattace Raso G. Simeoli R. Russo R. Iacono A. Santoro A. Paciello O. (2013). Effects of sodium butyrate and its synthetic amide derivative on liver inflammation and glucose tolerance in an animal model of steatosis induced by high fat diet. *PLoS One* 8:e68626. 10.1371/journal.pone.0068626 23861927 PMC3702592

[B32] Moeller A. H. Sanders J. G. (2020). Roles of the gut microbiota in the adaptive evolution of mammalian species. *Philos. Trans. R. Soc. London Ser. B Biol. Sci.* 375:20190597. 10.1098/rstb.2019.0597 32772670 PMC7435157

[B33] Mollica M. P. Raso M. G. Cavaliere G. Trinchese G. De Filippo C. Aceto S. (2017). Butyrate regulates liver mitochondrial function, efficiency, and dynamics in insulin-resistant obese mice. *Diabetes* 66 1405–1418. 10.2337/db16-0924 28223285

[B34] Morrison D. J. Preston T. (2016). Formation of short chain fatty acids by the gut microbiota and their impact on human metabolism. *Gut Microbes* 7 189–200. 10.1080/19490976.2015.1134082 26963409 PMC4939913

[B35] Mu Y. Ma Z. Q. Zhu W. L. Zhang D. Wang Z. K. (2015). Study of body mass, thermogenesis and relative fatness in *Eothenomy miletus* from yunnan Province. *J. Yunnan Normal Univ.* 35 58–63. 10.1080/11250003.2014.902511

[B36] Nagy T. R. Negus N. C. (1993). Energy acquisition and allocation in male collared lemmings (*Dicrostonyx groenlandicus*): Effects of photoperiod, temperature, and diet quality. *J. Mammal.* 74:990. 10.2307/1382438

[B37] Ni J. Tokuda G. (2013). Lignocellulose-degrading enzymes from termites and their symbiotic microbiota. *Biotechnol. Adv.* 31 838–850. 10.1016/j.biotechadv.2013.04.005 23623853

[B38] Nishihara K. van Niekerk J. Innes D. He Z. Cánovas A. Guan L. L. (2023). Transcriptome profiling revealed that key rumen epithelium functions change in relation to short-chain fatty acids and rumen epithelium-attached microbiota during the weaning transition. *Genomics* 115:110664. 10.1016/j.ygeno.2023.110664 37286013

[B39] Ormerod K. L. Wood D. L. Lachner N. Gellatly S. L. Daly J. N. Parsons J. D. (2016). Genomic characterization of the uncultured Bacteroidales family S24-7 inhabiting the guts of homeothermic animals. *Microbiome* 4:36. 10.1186/s40168-016-0181-2 27388460 PMC4936053

[B40] Parker B. J. Wearsch P. A. Veloo A. C. M. Rodriguez-Palacios A. (2020). The genus *Alistipes*: Gut bacteria with emerging implications to inflammation, cancer, and mental health. *Front. Immunol.* 11:906. 10.3389/fimmu.2020.00906 32582143 PMC7296073

[B41] Pfannenberg C. Werner M. K. Ripkens S. Stef I. Deckert A. Schmadl M. (2010). Impact of age on the relationships of brown adipose tissue with sex and adiposity in humans. *Diabetes* 59 1789–1793. 10.2337/db10-0004 20357363 PMC2889780

[B42] Pu G. Hou L. M. Du T. R. Zhou W. D. Liu C. X. Niu P. P. (2023). Increased proportion of fiber-degrading microbes and enhanced cecum development jointly promote host to digest appropriate high-fiber diets. *mSystems* 8:e0093722. 10.1128/msystems.00937-22 36511688 PMC9948726

[B43] Pu G. Li P. H. Du T. R. Niu Q. Fan L. J. Wang H. (2020). Adding appropriate fiber in diet increases diversity and metabolic capacity of distal gut microbiota without altering fiber digestibility and growth rate of finishing pig. *Front. Microbiol.* 11:533. 10.3389/fmicb.2020.00533 32328041 PMC7160236

[B44] Qiu X. J. Qin X. L. Chen L. M. Chen Z. M. Hao R. K. Zhang S. Y. (2022). Serum biochemical parameters, rumen fermentation, and rumen bacterial communities are partly driven by the breed and sex of cattle when fed high-grain Diet. *Microorganisms* 10:323. 10.3390/microorganisms10020323 35208778 PMC8878564

[B45] Roediger W. E. (1982). Utilization of nutrients by isolated epithelial cells of the rat colon. *Gastroenterology* 83 424–429. 7084619

[B46] Russell W. Duthie G. (2011). Plant secondary metabolites and gut health: The case for phenolic acids. *Proc. Nutr. Soc.* 70 389–396. 10.1017/S0029665111000152 21781364

[B47] Salyers A. A. West S. E. Vercellotti J. R. Wilkins T. D. (1977). Fermentation of mucins and plant polysaccharides by anaerobic bacteria from the human colon. *Appl. Environ. Microbiol.* 34 529–533. 10.1128/aem.34.5.529-533.1977 563214 PMC242695

[B48] Scott K. P. Gratz S. W. Sheridan P. O. Flint H. J. Duncan S. H. (2013). The influence of diet on the gut microbiota. *Pharmacol. Res.* 69 52–60. 10.1016/j.phrs.2012.10.020 23147033

[B49] Sender R. Fuchs S. Milo R. (2016). Revised estimates for the number of human and bacteria cells in the body. *PLoS Biol.* 14:e1002533. 10.1371/journal.pbio.1002533 27541692 PMC4991899

[B50] Serena C. Ceperuelo-Mallafré V. Keiran N. Queipo-Ortuño M. I. Bernal R. Gomez-Huelgas R. (2018). Elevated circulating levels of succinate in human obesity are linked to specific gut microbiota. *ISME J.* 12 1642–1657. 10.1038/s41396-018-0068-2 29434314 PMC6018807

[B51] Steelman S. M. Chowdhary B. P. Dowd S. Suchodolski J. Janeèka J. E. (2012). Pyrosequencing of 16S rRNA genes in fecal samples reveals high diversity of hindgut microflora in horses and potential links to chronic laminitis. *BMC Vet. Res.* 8:231. 10.1186/1746-6148-8-231 23186268 PMC3538718

[B52] Tsuji Y. Hanya G. Grueter C. C. (2013). Feeding strategies of primates in temperate and alpine forests: Comparison of Asian macaques and colobines. *Primates* 54 201–215. 10.1007/s10329-013-0359-1 23708996

[B53] Van Dyke M. I. McCarthy A. J. (2002). Molecular biological detection and characterization of Clostridium populations in municipal landfill sites. *Appl. Environ. Microbiol.* 68 2049–2053. 10.1128/AEM.68.4.2049-2053.2002 11916731 PMC123838

[B54] Varel V. H. Dehority B. A. (1989). Ruminal cellulolytic bacteria and protozoa from bison, cattle-bison hybrids, and cattle fed three alfalfa-corn diets. *Appl. Environ. Microbiol.* 55 148–153. 10.1128/aem.55.1.148-153.1989 2705767 PMC184069

[B55] Veloso C. Bozinovic F. (1993). Dietary and digestive constraints on basal energy metabolism in a small herbivorous rodent. *Ecology* 74 2003–2010. 10.2307/1940843

[B56] Wang Q. Zhang T. B. Yi G. H. Chen T. Y. Bie X. J. He Y. X. (2017). Tempo-spatial variations and driving factors analysis of net primary productivity in the Hengduan mountain area from 2004 to 2014. *Acta Ecol. Sin.* 37 3084–3095.

[B57] Xiao L. Feng Q. Liang S. S. Sonne S. B. Xia Z. K. Qiu X. M. (2015). A catalog of the mouse gut metagenome. *Nat. Biotechnol.* 33 1103–1108. 10.1038/nbt.3353 26414350

[B58] Yan B. W. Zhu W. L. (2023). Research on feeding habits and stomach fungi in *Eothenomys miletus* from Hengduan mountain regions. *Life Res.* 6:11. 10.17520/biods.2001011 34063014

[B59] Yan B. W. Jia T. Wang Z. K. Zhu W. L. (2022). Comparative research of intestinal microbiota diversity and body mass regulation in *Eothenomys miletus* from different areas of Hengduan mountain regions. *Front. Microbiol.* 13:1026841. 10.3389/fmicb.2022.1026841 36325022 PMC9619095

[B60] Zhang Z. G. Xu D. M. Wang L. Hao J. J. Wang J. F. Zhou X. (2016). Convergent evolution of rumen microbiomes in high-altitude mammals. *Curr. Biol.* 26 1873–1879. 10.1016/j.cub.2016.05.012 27321997

[B61] Zhao Z. J. Wang D. H. (2007). Effects of diet quality on energy budgets and thermogenesis in Brandt’s voles. *Comp. Biochem. Physiol.* 148A 168–177. 10.1016/j.cbpa.2007.04.001 17482858

[B62] Zhu W. L. Cai J. H. Lian X. Wang Z. K. (2011). Effects of photoperiod on energy intake, thermogenesis and body mass in *Eothenomys miletus* in Hengduan Mountain region. *J. Thermal Biol.* 36 380–385. 10.1016/j.jtherbio.2011.06.014

[B63] Zhu W. L. Jia T. Lian X. Wang Z. K. (2010). Effects of cold acclimation on body mass, serum leptin level, energy metabolism and thermognesis in *Eothenomys miletus* in Hengduan Mountains region. *J. Thermal Biol.* 35 41–46. 10.1016/j.jtherbio.2009.10.006

[B64] Zhu W. L. Zhang H. Zhang L. Yu T. T. Wang Z. K. (2014). Thermogenic properties of Yunnan red-backed voles (*Eothenomys miletus*) from the Hengduan mountain region. *Anim. Biol.* 64 59–73. 10.1163/15707563-00002430

